# Construction and Characterization of Immortalized Skin Fibroblasts from Milu Deer

**DOI:** 10.3390/ani15192889

**Published:** 2025-10-02

**Authors:** Pan Zhang, Riujia Liu, Zhenyu Zhong, Yunfang Shan, Zhibin Cheng, Qingyun Guo, Hao Zhang, Frank Hailer, Jiade Bai

**Affiliations:** 1 Beijing Milu Ecological Research Center, Beijing Academy of Science and Technology, Beijing 100076, China; zhangpan95@163.com (P.Z.); s20233040754@cau.edu.cn (R.L.); miluhome@126.com (Z.Z.); shanyunfang@yeah.net (Y.S.); czb@milupark.org.cn (Z.C.); guoqingyun1987@126.com (Q.G.); 2 Organisms and Environment, School of Biosciences, Cardiff University, Cardiff CF10 3AT, UK; 3 State Key Laboratory of Animal Biotech Breeding, Beijing Key Laboratory for Animal Genetic Improvement, College of Animal Science and Technology, China Agricultural University, Beijing 100193, China; zhanghao827@163.com; 4 Cardiff University & Institute of Zoology Joint Laboratory for Biocomplexity Research (CIBR), Chinese Academy of Sciences, Beijing 100101, China

**Keywords:** Milu deer, fibroblast cell line, SV40T, vimentin

## Abstract

**Simple Summary:**

The Milu deer, also known as Père David’s deer, is an endangered species native to China. To contribute to ongoing conservation efforts, we established an immortalized cell line from the skin of a male fawn. Sustained cell division in vitro was achieved through introduction of the SV40T gene. Further, we optimized culturing conditions for growing these cells, and confirmed that cells maintain the essential characteristics of normal skin fibroblasts, with a strong capacity for cell proliferation. The availability of this immortal cell line provides a novel, valuable resource for future scientific research and conservation efforts aimed at protecting Milu deer.

**Abstract:**

Somatic cell preservation is an effective strategy for conserving the genetic potential of endangered species. To contribute to the conservation of the Milu deer (*Elaphurus davidianus*), this study aimed to establish and characterize an immortalized skin fibroblast cell line (ML-iSFC). The cell line is based on fibroblasts from the skin tissue of a male fawn of Milu deer. Optimal culture conditions were determined by supplementing the culture medium with different growth factors, and immortalization was achieved through simian virus 40 large T antigen (SV40T) transduction. Optimal culturing conditions for the cells were determined by adding a range of growth factors. The cellular morphology, growth characteristics, and marker expression of the cells were further evaluated. Cell cycle and proliferation were assessed by flow cytometry and CCK-8 assays, respectively. Chromosomes were determined by karyotype analysis. The highest cell growth rate was observed when the culture medium was supplemented with 3 ng/mL of FGF2. The fibroblast-specific marker vimentin (VIM) was expressed in both ML-SFC and ML-iSFC, while the epithelial marker keratin 18 (KRT18) was weakly expressed in ML-SFC cells. Cell proliferation and cell-cycle analysis revealed that ML-iSFC exhibited a higher growth rate and greater vitality compared to ML-SFC. Karyotype analysis showed that ML-iSFC maintained the same chromosome number and morphology as ML-SFC. In summary, this study reports the successful construction of an immortalized fibroblast cell line from Milu deer, which will serve as a valuable tool for Milu deer conservation.

## 1. Introduction

Biodiversity has long been affected by numerous anthropogenic factors, leading many species to face instability and the threat of extinction. Given the importance of biodiversity for human well-being and ecosystem functioning, measures are taken globally to protect species and ecosystems. Among these, the conservation of animal genetic resources is considered an effective and promising strategy [1]. Compared to reproductive cells and embryos, the cryopreservation of somatic cells is considered a promising method for germplasm conservation, especially for critically endangered species and precious animals that are difficult to access or have already died [2,3,4]. Somatic cells provide a viable, practical, and timely backup of genetic material, while also addressing the challenges and limitations of animal experimentation.

Skin fibroblasts are the most commonly used type of somatic cells in somatic cell cloning. Many species have been produced using somatic cell cloning techniques, such as sheep [5], goats [6], cattle [7] and pigs [8]. In addition, fibroblast cell lines can serve as feeder cells for the derivation and maintenance of stem cells. These cells play a crucial role in maintaining skin function, actively participating in wound healing processes and serving as sentinel cells through the synthesis of chemokines and the regulation of inflammation [9,10]. In recent years, research on the induction of pluripotent stem cells from fibroblasts has been conducted across multiple species [11,12,13]. Overall, fibroblasts are indispensable materials for the conservation of endangered species and also play a crucial role in research in fields such as genetics, pathology, and toxicology. However, the limited proliferative and differentiation capacities of primary fibroblasts, along with their relatively short passage lifespan, hinder their widespread application. The currently available methods for cell immortalization, such as simian virus 40 large T antigen (SV40T) or EBV-mediated transformation and overexpression of human telomerase reverse transcriptase (hTERT), have substantially increased the in vitro lifespan of various human cell types [14,15,16].

In this study, we aimed to establish an immortalized skin fibroblast cell line of the Chinese Milu deer, also known as Père David’s deer (*Elaphurus davidianus*), by transducing primary Milu skin fibroblasts with SV40T. The species is listed in the IUCN Red List as extinct in the wild, and listed as a national first-level protected wild species in China. While some previous reports have described the preservation of genetic materials for Milu deer, none included immortalized cell lines. Our present study therefore aimed to isolate and establish an immortalized Milu skin fibroblast cell line, to provide a viable and scalable source of genetic material and living cells, and offering various avenues for fundamental molecular research on Milu conservation [17].

## 2. Materials and Methods

### 2.1. Tissue Collection

Skin tissue was collected from a male Milu deer fawn that had starved to death at Beijing Milu Ecological Research Center, after being abandoned by its mother. The tissue was transported to the laboratory in Dulbecco’s Modified Eagle Medium (DMEM, GIBCO, Grand Island, NY, USA), supplemented with 2% antibiotics Penicillin-Streptomycin (200 U/mL penicillin and 200 µg/mL streptomycin, Sigma, Beijing, China). All experimental guidelines were approved by the Committee on the Ethics of Animal Experiments of the Beijing Milu Ecological Research Center (permit number: 2024-004).

### 2.2. Primary Culture and Subculture

The skin tissue was disinfected by soaking in 75% alcohol for 10 min and then washed three times with PBS. Using sterile surgical scissors, the tissue was cut into 1 mm^2^ pieces and digested with 1% trypsin-EDTA (GIBCO, Grand Island, NY, USA) for 20 min. The tissue was then further cut with sterile surgical scissors. Following this, the minced tissue fragments were digested with collagenase type II (Invitrogen, Carlsbad, CA, USA) at 37 °C for 2 h. Then, DMEM complete culture medium (containing 10% fetal bovine serum, 100 U/mL penicillin, and 100 U/mL streptomycin) was added to terminate the digestion. Thereafter, the digested tissue fragments were seeded into 75-cm^2^ cell culture flasks and cultured in DMEM complete culture medium at 37 °C in an atmosphere of 5% CO_2_.

When the confluence of the cells in the culture flask reached 80%, the culture medium was discarded, and the cells were washed twice with PBS. Then, 0.2% trypsin-EDTA (GIBCO, Grand Island, NY, USA) was added to digest the cells for 5 min. The digestion was terminated by adding DMEM complete culture medium, and the cell suspension was collected. The cell suspension was centrifuged at 1000 rpm for 5 min. The supernatant was then removed, and the pellet was resuspended in fresh culture medium. Finally, the cell suspension was transferred to new culture dishes at a 1:3 split ratio for subculturing.

### 2.3. Lentivirus Production and Target Cell Transduction

SV40T stable lentivirus was purchased from Genomeditech (Shanghai, China) Co. Ltd. The lentivirus was obtained by co-transfecting a mixture of GM easy^TM^ Lentiviral Mix, HG Transgene^TM^ Reagent, and the pGMLV-EF1a-SV40T-Puro plasmid into HEK-293T cells. For transduction, the cells were seeded in a 12-well plate. Then, 50 µL of lentivirus (viral titer of 10^7^ TU/mL, MOI = 50) was added to each well, and the cells were cultured in DMEM complete culture medium containing 5 µg/mL polybrene. After 48 h, the medium was replaced with medium containing 2 µg/mL puromycin (Sigma, Beijing, China), to select for stably transduced cells. The medium was changed every two days, and once all negative control cells had died, the surviving cells were passaged and further selected: After two consecutive passages, the immortalized Milu skin fibroblast cell line was obtained.

### 2.4. Immunofluorescence Staining

Immunofluorescence analysis was used to detect the expression of Vimentin (VIM), keratin 18 (KRT18) and SV40 large T antigen (SV40T) in fibroblasts, with MAC-T serving as the positive control for keratin. The steps are as follows: Fix the cells with 4% paraformaldehyde for 10 min, and then permeabilize with 1% Triton X-100 for 1 h. Subsequently, rabbit anti-vimentin protein (1:400 dilution; ABclonal Biotechnology Co., Ltd., Wuhan, China), rabbit anti-keratin primary antibody (1:400 dilution; ABclonal Biotechnology Co., Ltd.), rabbit anti-SV40T antigen primary antibody (1:200 dilution; AbMart Bio-tech Co. Ltd., Shanghai, China), and mouse anti-α-tubulin primary antibody (1:500 dilution; ABclonal Biotechnology Co., Ltd.) were added separately and incubated overnight at 4 °C. Next, the secondary antibody (Cy3-conjugated Goat anti-Rabbit IgG (H + L), 1:500 dilution; ABclonal Biotechnology Co., Ltd.; ABflo^®^ 488-conjugated goat anti-Mouse IgG (H + L) (1:500 dilution; ABclonal Biotechnology Co., Ltd.) was added and incubated at room temperature in the dark for 1 h. The samples were washed with PBS five times, during which 4′,6-diamidino-2-phenylindole (DAPI, Solarbio Science & Technology Co., Ltd., Beijing, China) was added for 10 min. Finally, cells were observed under a fluorescence microscope (ZEISS, Jena, Germany).

### 2.5. Western Blot

The protein concentration was quantified using a BCA protein assay kit (Thermo Fisher Scientific, Waltham, MA, USA). Subsequently, 20 µg of total protein from each sample was loaded onto an SDS-PAGE gel for electrophoretic separation, followed by transfer to a 0.45 µm Polyvinylidene fluoride (PVDF) membrane. After blocking with 5% BSA for 1 h, the membrane was incubated overnight at 4 °C with primary antibodies, including abbit anti-vimentin antibody (1:2000 dilution; ABclonal Biotechnology Co., Ltd.), rabbit anti-keratin antibody (1:1000 dilution; ABclonal Biotechnology Co.), rabbit anti-GAPDH antibody (1:4000 dilution; ABclonal Biotechnology Co.), rabbit anti-SV40T antigen–antibody (1:200 dilution; AbMart Bio-tech Co. Ltd., Shanghai, China). After washing the primary antibody with TBST three times, the membrane was incubated with HRP-conjugated goat anti-rabbit secondary antibody (1:2000 dilution; Abcam, Cambridge, UK) at room temperature for 2 h. After washing five times with TBST, the membrane was developed using a chemiluminescent substrate and imaged using a gel imaging system (Tannon, Shanghai, China).

### 2.6. Karyotyping

Colchicine was added to both immortalized Milu skin fibroblasts (ML-iSFC, passaged to the 20th generation, P20) and primary Milu skin fibroblasts (ML-SFC, passaged to the 4th generation, P4) for 2.5 h, followed by incubation in a pre-warmed (37 °C) hypotonic 0.075 mol/L potassium chloride (KCl) solution for 15 min. The cells were then fixed with freshly prepared fixative (methanol:acetic acid = 3:1) for 30 min, with fixation repeated three times. A small volume of cell suspension was pipetted, and allowed to drop gradually from a height of 30–40 cm onto microscope slides. The cells on the slides were heat-fixed by passing each slide repeatedly through the flame of an alcohol lamp. The slides were then incubated at 75 °C for 3 h, treated with trypsin solution (Gibco, Grand Island, NY, USA) for 20–25 s, washed briefly with physiological saline, and immediately placed into pre-warmed (37 °C) Giemsa staining solution for staining (Sigma, Beijing, China. After rinsing with distilled water, the slides were examined under an Olympus CX41 microscope (Olympus Corporation, Tokyo, Japan), and images were acquired and analyzed using METAFER software (version 4.3, MetaSystems, Altlussheim, Germany).

### 2.7. Growth Curve Analysis

ML-iSFC (P20) and ML-SFC (P4) were seeded at 1 × 10^3^ cells per well in 96-well plates. Cell viability was measured every 24 h for 14 days using the CCK-8 assay (Beyotime Biotechnology, Shanghai, China), with 10 replicates per sample. Then, 10 μL of CCK-8 solution and 190 μL of complete cell culture medium were added to each well. After incubating for 1 h in a cell incubator, the absorbance at 450 nm was measured using a microplate reader (Biotek, Winooski, VT, USA), and the cell proliferation kinetics curve was plotted.

### 2.8. Cell-Cycle Detection

Cells were harvested by digestion with 0.2% trypsin when the cell convergence reached 70%~80%. Then, pre-chilled 70% ethanol at −20 °C was added for fixation, and the cells were incubated overnight at 4 °C. The staining working solution was prepared by mixing the RNase A stock solution (100 μg/mL) and the PI staining solution (50 μg/mL) at a volume ratio of 1:4. The cells were washed twice with PBS, then stained with 500 µL of the pre-prepared PI/RNase A staining working solution at room temperature, protected from light, for 30–60 min. The samples were then analyzed, and red fluorescence was recorded at the excitation wavelength of 488 nm.

### 2.9. Statistical Analysis

The protein relative expression levels were analyzed and graphed using GraphPad Prism 9. All results are presented as mean ± standard error (SE), and statistical analysis was performed using unpaired two-tailed *t*-tests.

## 3. Results

### 3.1. Culture and Cryopreservation of Primary Milu Skin Fibroblast Cells (ML-SFC)

**Figure 1:**
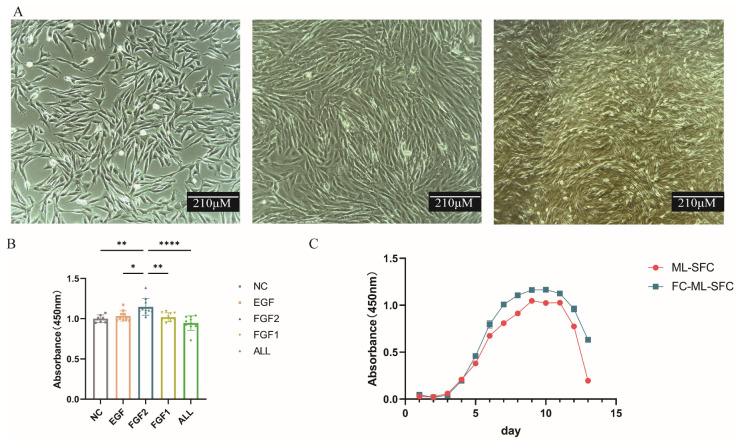
Isolation and culture of primary Milu skin fibroblasts (ML-SFC). (**A**) Cell Morphology of ML-SFC (from left to right, 24 h, 36 h, 48 h after being seeded). (**B**) Effect of Growth Factor Supplementation on Cell Proliferation Capacity. (**C**) Cell Growth Curves Before and After Cryopreservation. ML-SFC: Primary Milu skin fibroblast cells. FC-ML-SFC: Frozen cells-primary Milu skin fibroblast cells. EGF: epidermal growth factor. FGF1: fibroblast growth factor 1. FGF2: fibroblast growth factor 2. * *p* < 0.05, ** *p* < 0.01, **** *p* < 0.00001.

The isolated ML-SFC are shown in Figure 1. The cells are clearly visible and exhibit a fibroblast-like morphology with a monolayer arrangement. The cell shape is primarily spindle-like, and the cells are relatively elongated. When grown densely, the cells are arranged in a relatively orderly manner (Figure 1A). We added three types of growth factors to the complete medium: epidermal growth factor (EGF, 10 ng/mL), fibroblast growth factor 1 (FGF1, 1 ng/mL), and fibroblast growth factor 2 (FGF2, 3 ng/mL). We found that when FGF2 was added alone to the medium, the fibroblasts grew at the fastest rate. When all three growth factors were added together (EGF: FGF1: FGF2 = 10:1:3), the growth rate was the slowest (Figure 1B). Furthermore, we assessed cell viability before and after cryopreservation and found that the cell proliferation rate during the exponential growth phase was higher post-thaw than prior to freezing. This indicates that the cryopreservation process did not impair cell proliferation, and may have even enhanced the proliferative capacity of the cells (Figure 1C).

### 3.2. Identification of ML-SFC

**Figure 2:**
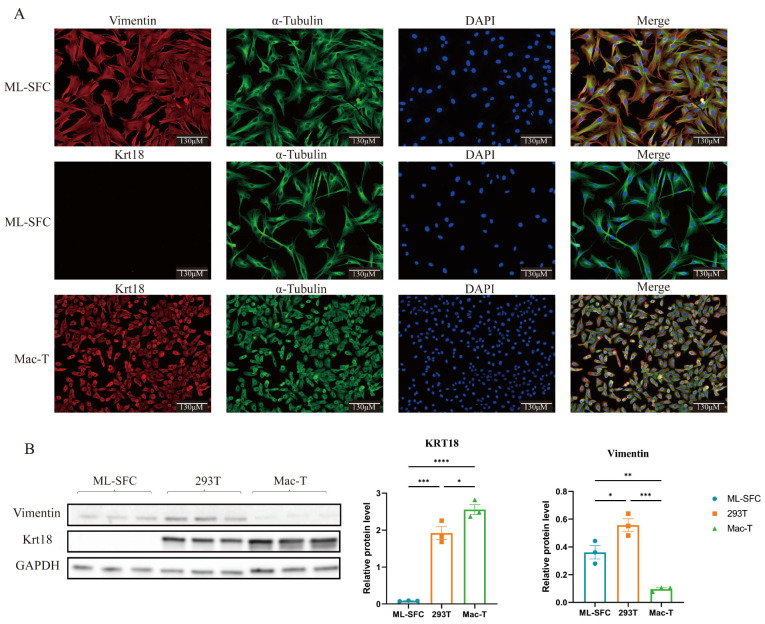
Identification of ML-SFC. (**A**) Immunofluorescence analysis of vimentin and keratin 18 expression in ML-SFC. (**B**) Western blot analysis of vimentin and keratin 18 expression in ML-SFC. Data represent mean ± SE. * *p* < 0.05, ** *p* < 0.01, *** *p* < 0.001, **** *p* < 0.0001.

To further confirm that the isolated ML-SFC are fibroblasts, we performed immunofluorescence staining and Western blotting to examine the expression of specific markers for fibroblasts and epithelial cells, namely vimentin (VIM) and keratin 18 (KRT18). As shown in Figure 2, VIM was highly expressed exclusively in ML-SFC, while KRT18 was expressed in the epithelial-like Mac-T cells. Western blot analysis revealed the same results. These findings indicate that the isolated ML-SFC are pure fibroblasts and do not contain epithelial cells.

### 3.3. Establishment of Immortalized Milu Skin Fibroblasts (ML-iSFC)

**Figure 3:**
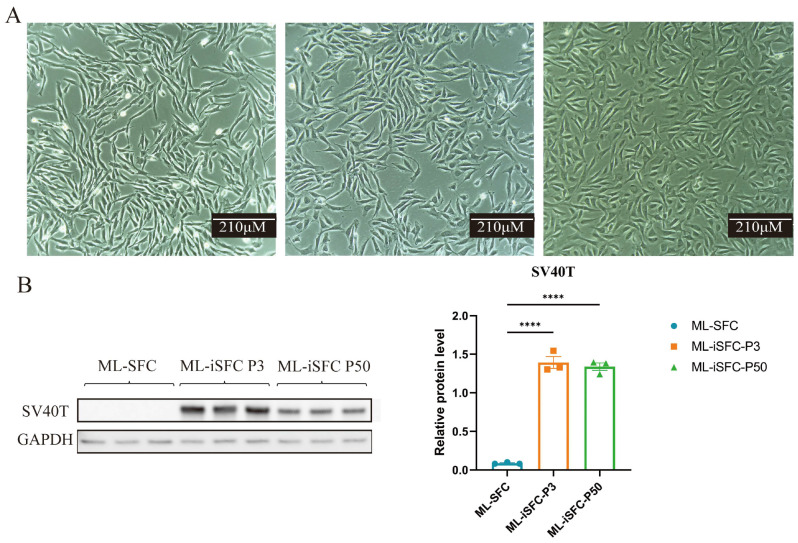
(**A**) Morphology of different passages of ML-iSFC cells after introduction of the exogenous SV40T gene. From left to right: ML-SFC, ML-iSFC at passage 3, and ML-SFC at passage 50. (**B**) Western blot showed that SV40T is expressed in ML-iSFC cells of different passages. P3: passaged to the third generation, P50: passaged to the 50th generation. **** *p* < 0.0001.

In this study, the exogenous SV40T gene was introduced to promote cell immortalization. After selection with puromycin, ML-SFC were successfully passaged beyond 50 generations. As shown in Figure 3A, following the introduction of the exogenous SV40T gene, the cells overcame the division limit and continued to proliferate. The ML-iSFC exhibited typical long, spindle-shaped morphology with uniform cell appearance in each passage. We analyzed the expression level of SV40T in the cells and found that SV40T is stably expressed in cells passed to the 50th generation (Figure 3B).

**Figure 4:**
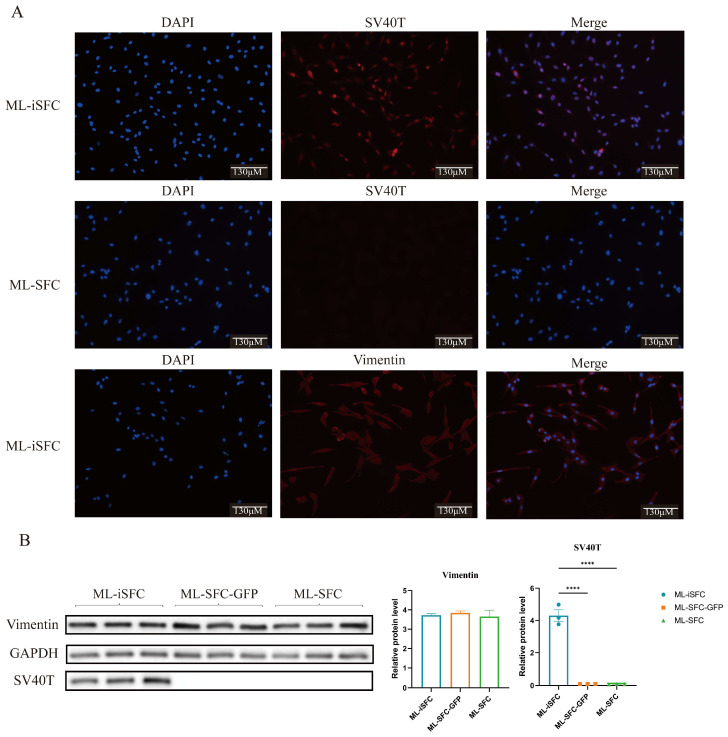
Immunofluorescence and Western blot analysis of vimentin and SV40T expression. (**A**) Immunofluorescence analysis of vimentin and SV40T expression in ML-iSFC. (**B**) Western blot analysis of vimentin and SV40T expression in ML-iSFC. ML-SFC-GSP: SFC transfected with a vector lacking the SV40T gene, serving as the control for SV40T transfection. **** *p* < 0.0001.

We performed immunofluorescence staining and Western blotting to detect the expression of fibroblast-specific markers, vimentin (VIM), and the SV40T in the transfected ML-SFC. As shown in Figure 4, the SV40T was stably expressed in the transfected ML-SFC, while VIM was highly expressed in all types of fibroblasts. Western blot analysis confirmed these findings, indicating that the construction of the ML-iSFC was successful.

### 3.4. Growth Characteristics Analysis of ML-iSFC Cell Lines

**Figure 5:**
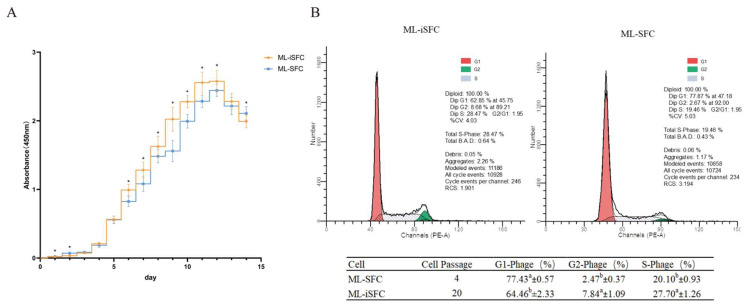
Cell growth curve plotting and cell-cycle detection. (**A**) The proliferation of SFC and iSFC cells was measured by CCK8 for 14 days. (**B**) Flow cytometry was used to assess the number of cells at different stages of the cell cycle in ML-SFC and ML-iSFC. The superscript lowercase letters a and b in table indicate a significant difference in different groups (*p* < 0.05). * *p* < 0.05.

To evaluate growth characteristics of the ML-iSFC cell line, we assessed the cell growth curves and cell cycle using CCK-8 assays and flow cytometry for both ML-SFC and SV40T-induced ML-iSFC cells. As shown in Figure 5, SFC exhibited an S-shaped growth curve. Compared to ML-SFC, ML-iSFC showed a faster growth rate and entered the growth plateau phase more rapidly (Figure 5A). Cell-cycle analysis revealed that the proportions of cells in the G2 and S phases were significantly higher in ML-iSFC than in ML-SFC (Figure 5B), indicating that ML-iSFC possesses a stronger proliferative capacity and a shorter cell cycle.

### 3.5. Karyotype Analysis

**Figure 6:**
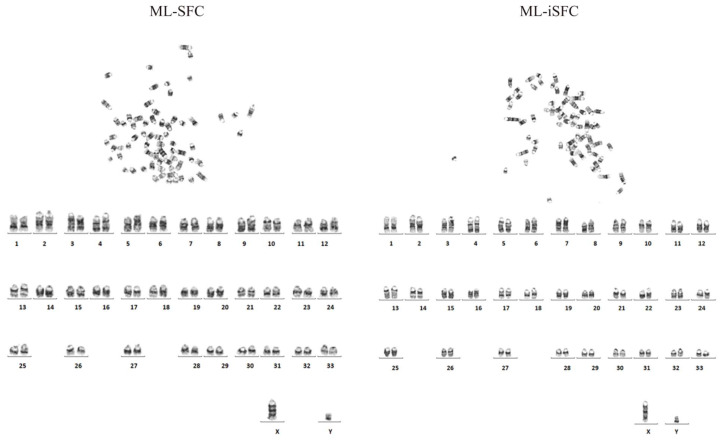
Comparison of karyotype analysis between ML-SFC (P4) and ML-iSFC (P20).

To assess whether ML-SFC immortalization induces chromosomal abnormalities, we performed karyotype analysis to examine the chromosome number and structure of ML-iSFC. As shown in Figure 6, compared to the control group ML-SFC, the immortalized ML-iSFC (P20) exhibited a normal Milu chromosome number (2n = 68) and structure, consistent with the normal chromosomal profile of ML-SFC, indicating that ML-iSFC maintains its normal biological characteristics.

## 4. Discussion

In this study, primary Milu Skin Fibroblast cells (ML-SFC) were successfully isolated and purified. Following characterization, ML-SFC were immortalized through SV40T transfection, yielding ML-iSFC. Compared to ML-SFC, ML-iSFC exhibited a significantly accelerated proliferation rate. Notably, unlike primary cells with limited passage capacity, ML-iSFC maintained robust proliferation without senescence or reduced growth kinetics up to passage 50, accompanied by stable SV40T expression and normal chromosomal structure and diploid number (2n = 68). The generation of an immortalized fibroblast cell line establishes a renewable in vitro platform for studying the species’ cellular mechanisms and preserving its somatic genetic material, supporting both research and conservation purposes. One direct application of the established cell line is its use in advancing assisted reproductive technologies for Milu deer.

Primary cells exhibit limited passaging capacity, with their maximum passage numbers varying significantly across species and tissue origins. While some cell lines can be subcultured for 3 passages [18], others can sustain 5–6 passages [15]. In our study, primary Milu skin fibroblast cells at passage 6 (P6) demonstrated significant morphological alterations, including cellular flattening, vacuolization, decreased proliferation rates, and impaired adherent properties. Following SV40T transduction, the cells retained normal morphology and proliferation potential through 50 passages. Previous studies have demonstrated that the SV40T gene alone is sufficient to immortalize mammalian cells [19,20,21,22]. Its core mechanism involves targeted inhibition of the tumor suppressor proteins p53 and the retinoblastoma protein (pRb) family, thereby bypassing G1/S arrest and promoting cell-cycle progression [23]. This allows cells to circumvent replicative senescence and the crisis phase, acquiring the capacity for unlimited proliferation [23,24,25,26]. Our flow cytometry analysis revealed that the higher proportion of cells in the G2 and S phases of the ML-iSFC cell cycle is highly consistent with this mechanism. We demonstrated the rapid proliferative capacity of the cells through cell proliferation curves. The fact that Western blot analysis detected sustained expression of SV40T in P50 cells further supports the successful establishment of an immortalized Milu cell line. The high expression of the fibroblast marker vimentin (VIM) and the near absence of the epithelial cell marker keratin 18 (KRT18) indicate the high purity of the cell line [27,28]. Notably, SV40T-mediated immortalization did not induce significant deviations in fundamental cellular properties. Karyotype analysis revealed that ML-iSFC cells at passage 50 (P50) maintained a normal diploid chromosome number (2n = 68) and structural integrity, with no detectable chromosomal aberrations, indicating preserved genomic stability during extended in vitro culture.

This study successfully established and verified an immortalized ML-iSFC. However, certain limitations remain. The current investigation only assessed the cells up to passage 50. Further characterization of higher passages is necessary to thoroughly validate the stability and sustained immortalization properties of this cell line. Additionally, the establishment of a cell line from a single individual provides only limited resources for Milu deer conservation efforts, while cell lines from multiple individuals would provide a more comprehensive bank of genetic material.

## 5. Conclusions

This study reports the successful establishment of the first immortalized skin fibroblast cell line (ML-iSFC) from the endangered Milu deer (*Elaphurus davidianus*) through SV40 large T antigen transduction. This cell line is highly homogenous with no detected contamination from epithelial cells, and it maintains rapid proliferation after 50 passages in vitro. Furthermore, supplementation with 3 ng/mL EGF2 in the culture medium markedly enhances ML-iSFC growth. The establishment of this cell line facilitates future research in vitro, and contributes to the preservation of Milu deer genetic resources.

## Data Availability

The ML-iSFC cell line is available to all researchers. Please contact the first author.
